# P-349. Using Participatory Design to Establish a Workflow for Administration of Long-Acting Cabotegravir + Rilpivirine in Community Pharmacies

**DOI:** 10.1093/ofid/ofaf695.567

**Published:** 2026-01-11

**Authors:** Kristen A Berg, Morgan K Morelli, Ashley M Hughes, Alexander Nelson, Luke Forkapa, Joshua Maierhofer, Margaret Oblak, Demetria Webb, Nicholas Riley, Melissa O Jenkins, Ann A Avery, Corrilynn O Hileman

**Affiliations:** Case Western Reserve University School of Medicine at The MetroHealth System, Cleveland, Ohio; MetroHealth Medical Center, Cleveland, Northern Mariana Islands; Case Western Reserve University School of Medicine at The MetroHealth System, Cleveland, Ohio; The MetroHealth System, Case Western Reserve University School of Medicine, Cleveland, Ohio; The MetroHealth System, Cleveland, Ohio; The MetroHealth System, Cleveland, Ohio; The MetroHealth System, Cleveland, Ohio; The MetroHealth System, Cleveland, Ohio; The MetroHealth System, Case Western Reserve University School of Medicine, Cleveland, Ohio; MetroHealth, Cleveland, Ohio; Case Western Reserve University School of Medicine, Cleveland, Ohio; The MetroHealth System, Case Western Reserve University School of Medicine, Cleveland, Ohio

## Abstract

**Background:**

Long acting cabotegravir and rilpivirine (LA CAB/RPV), the first fully injectable antiretroviral therapy for people with HIV (PWH), is administered intramuscularly typically by a nurse in a clinic. Limitations to this approach include staff availability, office hour restrictions, travel distance, and HIV stigma. Alternate models of care are needed to address these obstacles expanding access to LA CAB/RPV. We used a human-centered participatory approach to co-design a workflow adapting in clinic administration of LA CAB/RPV to community pharmacies.

Workflow for Long-acting Cabotegravir/Rilpivirine Administration in Community Pharmacies

**Figure:**
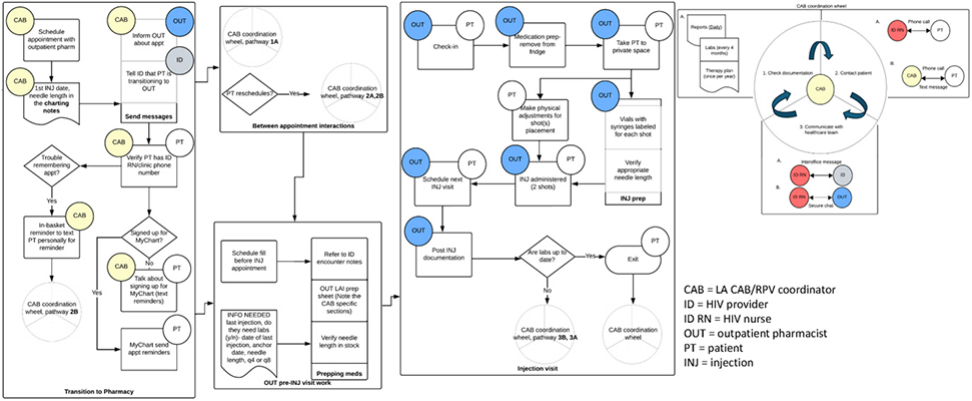

**Methods:**

Our design team consisted of 12 members, including HIV providers, pharmacists, nurses, informaticists, and PWH receiving LA CAB/RPV. The team met weekly for 5 sessions to delineate current practices and iteratively produce a modified workflow. We assessed barriers and facilitators of co-design through semi-structured interviews with design team members. A phenomenologically informed codebook thematic analysis identified key themes across interviews.

**Results:**

Factors identified as essential to the in clinic model of LA CAB/RPV administration included care coordination utilizing electronic health record reports to track patient follow-up, communication among the team and with the patient, and injection visit experience. The figure shows the workflow adapted to community pharmacies. Next, from interviews with 6 design team members, we identified 4 themes of successful co-design. First, meeting modality created essential infrastructure for collaboration with in-person interactions enabling more effective engagement. Second, strategic leadership shaped the process with atomization of a complex workflow into manageable steps which involved work between meetings to frame and organize discoveries. Third, team composition and inclusivity enriched the process where multidisciplinary representation contributed critical insights. Last, collaborative humility fostered participation across power differentials aiding the integration of expertise from all participants.

**Conclusion:**

Using co-design, we were able to adapt the workflow for in clinic administration of LA CAB/RPV to community pharmacies. Structural, compositional and interpersonal team factors contributed to the successful co-design process.

**Disclosures:**

Melissa O. Jenkins, Meliisa Jenkins, Gilead Sciences, Inc.: Grant/Research Support Corrilynn O. Hileman, MD, MS, Gilead: Grant/Research Support|ViiV: Grant/Research Support

